# Enhanced Semantic Representation Learning for Sarcasm Detection by Integrating Context-Aware Attention and Fusion Network

**DOI:** 10.3390/e25060878

**Published:** 2023-05-30

**Authors:** Shufeng Hao, Jikun Yao, Chongyang Shi, Yu Zhou, Shuang Xu, Dengao Li, Yinghan Cheng

**Affiliations:** 1 College of Data Science, Taiyuan University of Technology, Taiyuan 030024, China; haoshufeng@tyut.edu.cn (S.H.); xushuang@tyut.edu.cn (S.X.); lidengao@tyut.edu.cn (D.L.); 2 Key Laboratory of Big Data Fusion Analysis and Application of Shanxi Province, Taiyuan 030024, China; 3 School of Economics and Management, China University of Petroleum (East China), Qingdao 266580, China; 2108040211@s.upc.edu.cn; 4 School of Computer Science and Technology, Beijing Institute of Technology, Beijing 100081, China; cy_shi@bit.edu.cn (C.S.); chengyinghan@bit.edu.cn (Y.C.)

**Keywords:** sarcasm detection, representation learning, context-aware attention, fusion network

## Abstract

Sarcasm is a sophisticated figurative language that is prevalent on social media platforms. Automatic sarcasm detection is significant for understanding the real sentiment tendencies of users. Traditional approaches mostly focus on content features by using lexicon, n-gram, and pragmatic feature-based models. However, these methods ignore the diverse contextual clues that could provide more evidence of the sarcastic nature of sentences. In this work, we propose a Contextual Sarcasm Detection Model (CSDM) by modeling enhanced semantic representations with user profiling and forum topic information, where context-aware attention and a user-forum fusion network are used to obtain diverse representations from distinct aspects. In particular, we employ a Bi-LSTM encoder with context-aware attention to obtain a refined comment representation by capturing sentence composition information and the corresponding context situations. Then, we employ a user-forum fusion network to obtain the comprehensive context representation by capturing the corresponding sarcastic tendencies of the user and the background knowledge about the comments. Our proposed method achieves values of 0.69, 0.70, and 0.83 in terms of accuracy on the Main balanced, Pol balanced and Pol imbalanced datasets, respectively. The experimental results on a large Reddit corpus, SARC, demonstrate that our proposed method achieves a significant performance improvement over state-of-art textual sarcasm detection methods.

## 1. Introduction

Sarcasm is a form of figurative language in which the literal meaning of the utterance differs from the author’s underlying intention [1]. This form of expression is commonly observed on social media platforms, such as Twitter and Reddit. Due to the figurative nature of text and the deliberate ambiguity of sarcasm, automatic sarcasm detection has become a challenging task in opinion mining and sentiment analysis [2].

Early works on automatic sarcasm detection primarily relied on salient content features, including n-gram, lexical, and pragmatic features [3]. Specifically, unigrams, sentiment lexicons, emoticons, user mentions, and incongruity have been considered explicit cues found in sentences that can improve the performance of textual sarcasm detection. Most approaches use traditional statistical learning methods to model these explicit features, such as the Support Vector Machines, Naive Bayes, and Decision Trees [4,5]. As deep learning techniques have obtained success in natural language processing, neural networks are also used to capture the above features automatically. For example, the convolutional neural network, Bi-LSTM, and attention mechanism have been employed to capture the salient features of sarcasm [6,7,8]. However, these models only focus on explicit features and neglect implicit features, which are used to indicate the ambiguity of sarcasm expressions.

**Figure 1:**
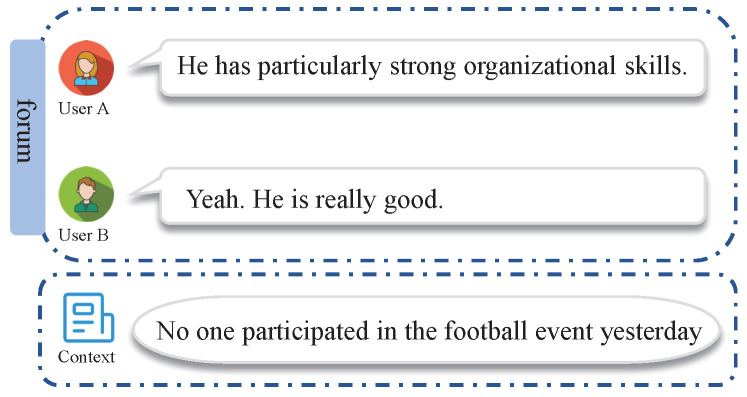
An toy example of sarcastic comments. The comments posted by users A and B are sarcastic and could be identified by the context provided by the contextual information.

To capture the implicit features of sarcasm expressions, contextual information has been adopted in later attempts at sarcasm detection. A toy example is provided in Figure 1 in order to figure out the context importance of sarcasm identification, where the comment samples were taken from a discussion forum. The comment *“He has particularly strong organizational skills.”* posted by user A and the comment *“Yeah. He is really good”* posted by user B do not seem to be sarcastic until the corresponding context *“No one participated in the football event yesterday”* is provided. Meanwhile, many forms of contextual dependencies, such as the commonsense context, conversational context, and user-specific context, have been explored by providing additional cues for sarcasm detection [3,9,10]. For example, contextual embedding is learned by using commonsense information from sentences, which is integrated with affective information for sarcasm detection [11,12]; the conversation context of an utterance is employed to capture the sarcastic nature of a conversation [13]; and historical data generated by users are adopted to capture a user’s sarcastic tendencies [1,9,14]. Additionally, topic contextual information is used to discover sarcasm-prevalent topics [3]. However, comment representation and context representation are not sufficiently integrated and interactive in these models, which degrades the sarcasm detection performance.

In this work, we explore a novel scenario involving the construction of an enhanced semantic representation learning network based on diverse contextual cues retrieved from the external discourses in social media for textual sarcasm detection, such as user profiling and forum topic information. Based on this, we propose a Contextual Sarcasm Detection Model (CSDM) by modeling enhanced semantic representation with user profiling and forum topic information, where context-aware attention and a user-forum fusion network are used to obtain the comment representation and context representation, respectively. Specifically, the model first employs diverse embedding methods to obtain textual comments and user and forum representations, which can provide the original situation in which comments occurred. Then, we employ a Bi-LSTM encoder and context-aware attention to obtain refined comment representation by capturing sentence composition information and the corresponding context situations. Then, we employ a user-forum fusion network to obtain the comprehensive context representation by capturing the corresponding sarcastic tendencies of the user and the background knowledge about the comments. Finally, a simple fusion strategy with comment and context representations is used to predict whether a comment is sarcastic or nonsarcastic. The main contributions of our work can be summarized as follows:

- A novel Contextual Sarcasm Detection Model is proposed that integrates enhanced semantic representation learning based on comment contents and diverse contextual clues;
- For comment representation learning, we employ a context-aware attention mechanism to capture the key parts of sentences in response to the corresponding contextual information;
- For context representation learning, we employ a user-forum fusion network to generate a comprehensive context representation by integrating user information and forum information;
- Experimental results from a large Reddit corpus, SARC, demonstrate that our proposed method achieves a significant performance improvement over state-of-art textual sarcasm detection methods.

## 2. Related Work

Sarcasm detection generally focuses on sarcasm identification from text, where sarcasm is a complex linguistic expression used on social media platforms. Previous works have employed representative indicators and discrete features to identify sarcasm expression. For example, Maynard et al. [15] proposed a hashtag tokeniser and demonstrated that hashtags can make sarcasm detection easy. Tony et al. [16] focused on humorous similes and employed corresponding simile patterns to predict ironic expressions. Lukin and Walker [17] applied n-gram features and syntactic patterns to create sarcasm indicators. Moreover, pragmatic factors, affective lexicons, semantic features, and incongruity features have been considered to improve the performance of sarcasm detection [3]. These studies mainly employed manual feature engineering, which is very time-consuming.

To capture comprehensive continuous features automatically, deep neural network approaches have been widely used for textual sarcasm detection. Soujanya et al. [6] employed a pretrained convolutional neural network to learn sentiment, emotion and personality features for sarcasm detection. Tay et al. [18] proposed a multidimensional intra-attention recurrent network to detect contrasting sentiments, situations and incongruities. Xiong et al. [7] developed a self-matching network based on a co-attention mechanism to capture sentence incongruities and incorporated a Bi-LSTM encoder to learn composition information. Pan et al. [19] used a self-attention weighted snippets model to detect the incongruity between sentence snippets. Akula and Garibay [8] employed a multihead self-attention method to identify crucial sarcastic cue words from the input and applied the recurrent units to learn long-range dependencies between these cue words. These works only focused on comments and neglected additional contextual information around the comments.

To improve the performance of sarcasm detection, contextual information has been incorporated. Zhang et al. [20] employed a gated pooling layer based on history tweets to obtain contextual features and combined this with a bi-directional gated recurrent neural network on targeted tweets in order to capture syntactic and semantic information. Du et al. [21] designed a dual-channel structure to effectively analyze the semantics of the target text and its sentimental context. Kamal and Abulaish [22] proposed a convolution and attention model with a bi-directional gated recurrent unit to detect self-deprecating sarcasm, where syntactic and semantic features and a comprehensive context representation were captured. Lou et al. [12] employed a graph convolutional network based on affective information and syntactical information from sentences in order to capture long-range incongruity patterns and inconsistent expressions in the context for sarcasm detection. Jena et al. [13] employed contextual information from sentences in a sequential manner for sarcasm detection. Baruah et al. [23] exploited the conversational context of the response and BERT to detect sarcasm. Wang et al. [24] explored a masking and generation paradigm in the context of extracting the context incongruities using an unsupervised perspective for sarcasm detection. Babanejad et al. [11] incorporated affective and contextual features to extend the architecture of BERT for sarcasm detection. Wen et al. [25] leveraged sememe knowledge and auxiliary information to learn the representations of the context and background of sarcasm expression. However, these methods neglect user information, which could provide support to improve the understanding of sarcastic tendencies in sentences and allow better sarcasm detection.

To capture the sarcastic tendencies of users for sarcasm detection, user embeddings are learned by considering the historic information of users on social media. For example, Amir et al. [9] learned user embeddings to capture homophily by projecting similar users into nearby regions of the embedding space and exploiting user embeddings with lexical representations of the content to recognize sarcasm. Plepi and Flek [1] applied a graph attention-based model to a social graph to explicitly model users’ social and historical contexts, where user embeddings with historic tweets were learned during sarcasm detection, similar to in the work of Amir [9]. However, these methods only focused on user embeddings and neglected common knowledge about the situations in which the comments were occurring. To solve this problem, Hazarika et al. [14] not only exploited user embeddings to encode the stylometric and personality features of users but also employed topic information from forums to capture the contextual information of comment discourses. The method employs a simple concatenation step to combine comment, user, and forum information, resulting in a poor performance. Thus, our proposed model applies enhanced semantic representation learning techniques based on context-aware attention and a user-forum fusion network, where contextual information is used to guide comment representation, and user and forum information is fused to learn the context representation.

## 3. Proposed Model

### 3.1. Overview

**Figure 2:**
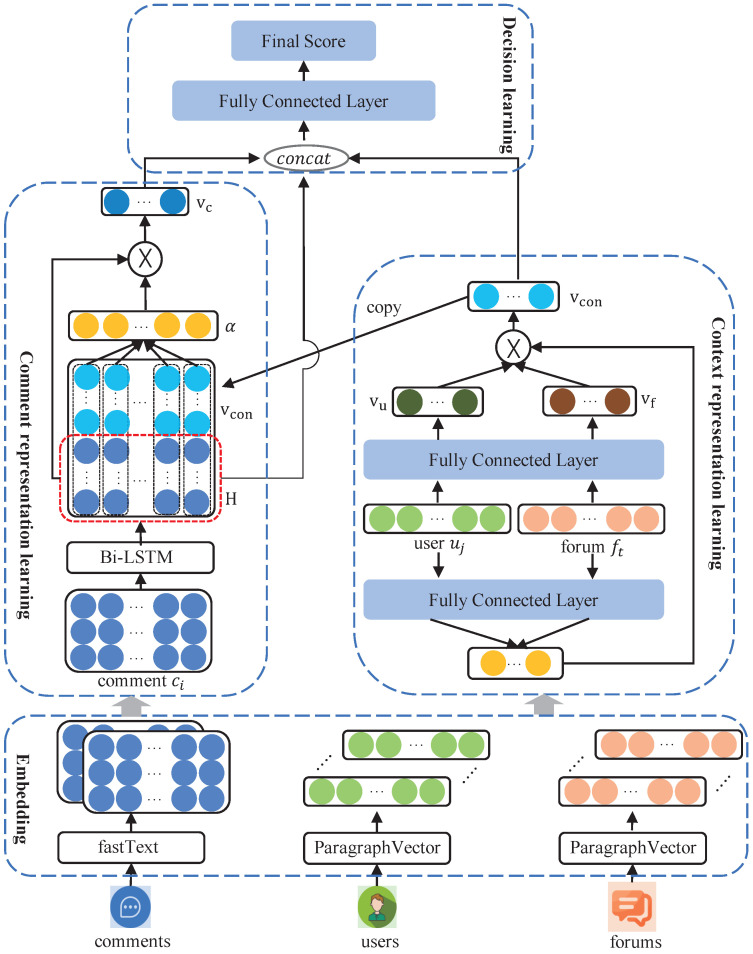
The framework of the proposed CSDM.

In this section, we illustrate the proposed Contextual Sarcasm Detection Model (CSDM) with enhanced semantic representation learning in detail. As demonstrated in Figure 2, the framework of the CSDM contains three parts: (1) comment representation learning, where Bi-LSTM is employed to obtain the sequential information of the comments, and context-aware attention is used to capture the comment representation comprehensively by considering the corresponding contextual information; (2) context representation learning, where a user-forum fusion network is used to obtain the context representation by integrating user and forum information; and (3) decision learning, where comments and contexts are concatenated by considering diverse semantic representations, and fully connected layers are employed to detect sarcasm.

### 3.2. Task Definition

Given the social media corpus SARC, the task is to identify sarcastic or nonsarcastic comments posted in specific topic forums. Each comment is accompanied by user details and parent comments, which could be used as contextual information. Mathematically, users in the corpus are denoted as $U=\{u_{1},\cdots ,u_{n}\}$, forums in the corpus are denoted as $F=\{f_{1},\cdots ,f_{m}\}$, and comments in the corpus are denoted as $C=\{c_{1},\cdots ,c_{t}\}$, where *n* is the number of users, *m* is the number of forums, and *t* is the number of comments. Therefore, our work aims to learn enhanced semantic representations by integrating context-aware attention and a fusion network in order to understand the sarcastic tendencies of comments comprehensively.

### 3.3. Comment Representation Learning

Comment representation learning aims to construct comprehensive comment representations. First, embedding techniques are employed to obtain the original comment representation with common contextual information. Then, a Bi-LSTM encoder is used to capture the word sequence order, and a context-aware attention mechanism is employed to obtain the key parts of comments.

Given the comment $c_{i}=\left[w_{1},\cdots ,w_{k}\right]$ containing *k* word *w* in social media, the pretrained fastText model [26] is used to generate comment embeddings $E\left(c_{i}\right)$. This is formulated as follows:

$$\begin{array}{c} E\left(c_{i}\right)=\mathrm{fastText}\left(c_{i}\right) \end{array}$$

The fastText model assigns a distinct vector representation to each word by capturing character n-gram information and word morphologies to obtain a fine-grained word representation.

To capture the sentence composition information of comments by considering the word sequence information, we employ the Bi-LSTM to obtain the hidden representations of comments $H=\left\{h_{1},\cdots ,h_{k}\right\}\in R^{d\times k}$ based on the comment embeddings $E\left(C\right)$, where *d* is the size of the hidden layers, and *k* is the sentence length. The details of the Bi-LSTM are as follows:

$$\begin{array}{cc} {\vec{h}}_{i} & =\vec{\mathrm{LSTM}}\left(E\left(w_{i}\right),{\vec{h}}_{i-1}\right)\\ {\overset{\leftarrow }{h}}_{i} & =\overset{\leftarrow }{\mathrm{LSTM}}\left(E\left(w_{i}\right),{\overset{\leftarrow }{h}}_{i-1}\right)\\ h_{i} & =\{{\vec{h}}_{i},{\overset{\leftarrow }{h}}_{i}\} \end{array}$$

where ${\vec{h}}_{i}$ and ${\overset{\leftarrow }{h}}_{i}$ represent the forward hidden state and the backward hidden state of the Bi-LSTM, respectively; $E\left(w_{i}\right)\in E\left(c_{i}\right)$ represents the ith word embedding vector of comment $c_{i}$.

Then, a context-aware attention mechanism is designed to capture the key parts of sentences in response to the corresponding contextual information. This was inspired by the work of Wang [27]. The attention mechanism can produce an attention weight vector α and a weighted hidden representation $v_{c}$. The details of the attention mechanism are as follows:

$$\begin{array}{cc} M & =\left[\begin{array}{c} H\\ v_{con}⊗e_{k} \end{array}\right]\\ \alpha & =\mathrm{softmax}(w^{T}M)\\ v_{c} & =H\alpha^{T} \end{array}$$

where $v_{con}\in R^{d_{c}}$, $M\in R^{(d+d_{c})\times k}$, $\alpha \in R^{k}$, and $v_{c}\in R^{d}$, and $d_{c}$ is the dimension of the context representation $v_{con}$ denoted in the following section. $w\in R^{\left(d+d_{c}\right)}$ is a projection parameter. α is a vector consisting of attention weights, and $v_{c}$ is the refined representation of the comment content within the corresponding given context. The operator ⊗ means $v_{con}⊗e_{k}=\left[v_{con};v_{con};\cdots ;v_{con}\right]$; that is, the operator repeatedly concatenates $v_{con}$*k* times, where $e_{k}\in R^{k}$ is a column vector with *k* 1s. The purpose of the operator $v_{con}⊗e_{k}$ is to create the same context embedding for each hidden representation from H. Furthermore, the weight vector α represents the important part of a sentence for the given contextual information.

Thus, comment representation learning can be used to obtain comprehensive comment representations by considering the effect of the corresponding contextual information, that is, the preliminary fusion between comment and context, where the contextual information is used to guide comment representation learning.

### 3.4. Context Representation Learning

For context representation learning, we employ a user-forum fusion network to generate the context representation for each corresponding comment sentence. Here, a user-forum fusion network is employed by integrating user information and forum information to construct the context situations in which comments occur in social media. User information can indicate the personal behaviors and interest tendencies of users, and forum information can provide background knowledge about the environment in which comments occur. Thus, user and forum information can provide evidence to capture the related sarcastic tendencies in the comments.

User embeddings and forum embeddings are first learned by using a pretrained model based on comment sentences posted by users on forums. Here, user embeddings are learned by considering the stylometric features of users, which are reflected by the accumulated historical posts of users. Meanwhile, forum embeddings are learned by considering comments belonging to a specific forum. First, a user corpus and a forum corpus are created for embedding learning. Comment sentences posted by each user in all forums are used to construct the user corpus, and comment sentences in a specific forum are employed to construct the forum corpus. We observe that the lengths of trained corpuses for context representation learning vary when the above data collection method is emplyed. Then, ParagraphVector [28] is used to generate user embeddings $E\left(U\right)$ and forum embeddings $E\left(F\right)$, since it is an unsupervised algorithm that learns fixed-length feature representations from variable-length pieces of text. Thus, user $u_{i}\in U$ and forum $f_{j}\in F$ can be represented as embedding vectors with different dimensions, i.e., $E(u_{i})$ and $E(f_{j})$, which are formulated as follows:

$$\begin{array}{c} E\left(u_{i}\right)=\mathrm{ParagraphVector}\left(u_{i}\right)\\ E\left(f_{j}\right)=\mathrm{ParagraphVector}\left(f_{j}\right) \end{array}$$

**Figure 3:**
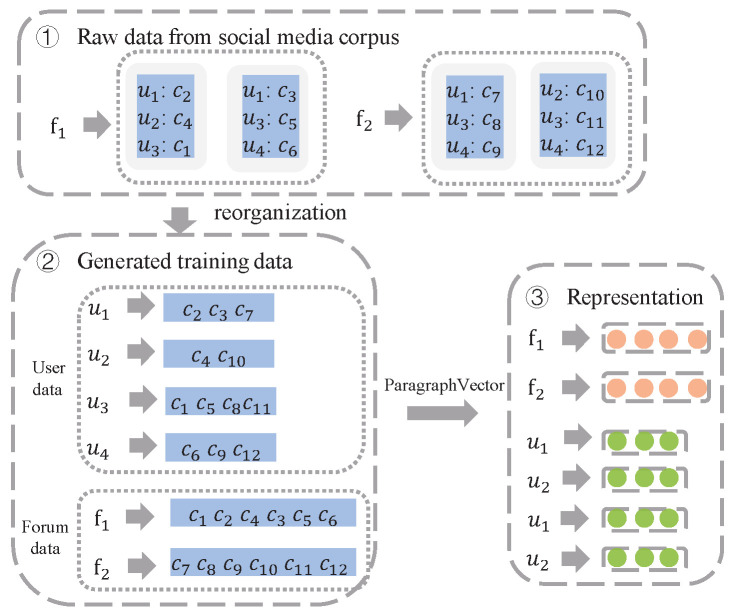
User representation and forum representation learning process. The process contains three parts: raw data from the social media corpus, generated training data, and the representation. For simplification, two forums ($f_{1}$ and $f_{2}$), four users ($u_{1}$, $u_{2}$, $u_{3}$ and $u_{4}$), and twelve comments ($c_{1},\cdots ,c_{12}$) are listed.

To clarify the learning process for user embeddings and forum embeddings, we show several samples in Figure 3 to detail the mechanism, where two forums ($f_{1}$ and $f_{2}$), four users ($u_{1},u_{2},u_{3}$ and $u_{4}$), and twelve comments ($c_{1},\cdots ,c_{12}$) are listed. It is shown that the process contains three parts: raw data from the social media corpus, generated training data, and the representation. In the example, we observe that the training datasets of users $u_{1},u_{2},u_{3}$, and $u_{4}$ are $\left\{c_{2},c_{3},c_{7}\right\}$, $\left\{c_{4},c_{10}\right\}$, $\left\{c_{1},c_{5},c_{8},c_{11}\right\}$, and $\left\{c_{6},c_{9},c_{12}\right\}$, respectively. Additionally, the training datasets of forums $f_{1}$ and $f_{2}$ are $\left\{c_{1},c_{2},c_{3},c_{4},c_{5},c_{6}\right\}$ and $\left\{c_{7},c_{8},c_{9},c_{10},c_{11},c_{12}\right\}$, respectively.

Considering the diverse aspects of context representation for sarcasm detection, we employ a user-forum fusion network to integrate user information and forum information into a fixed-length context vector, which is inspired by modality fusion [29]. First, the user embedding $E(u_{i})$ and forum embedding $E(f_{j})$ are transformed into a specific space with fixed-length forms $v_{u}$ and $v_{f}$. Then, a two-layer feed-forward neural network is employed to compute the normalized attention weights for the user and forum information. Finally, the context vector $v_{con}$ is obtained as the average weight of the transformed hidden representations $v_{u}$ and $v_{f}$. The mathematical formulations are as follows:

$$\begin{array}{cc} v_{u} & =\tanh\left(W_{u}\cdot E\left(u_{i}\right)+b_{u}\right)\\ v_{f} & =\tanh\left(W_{f}\cdot E\left(f_{i}\right)+b_{f}\right)\\ \alpha_{u} & =W_{u^{\prime \prime }}\cdot \tanh\left(W_{u^{\prime }}\cdot E\left(u_{i}\right)+b_{u^{\prime }}\right)+b_{u^{\prime \prime }}\\ \alpha_{f} & =W_{f^{\prime \prime }}\cdot \tanh\left(W_{f^{\prime }}\cdot E\left(f_{j}\right)+b_{f^{\prime }}\right)+b_{f^{\prime \prime }}\\ v_{con} & =\frac{e^{\alpha_{u}}}{e^{\alpha_{u}}+e^{\alpha_{f}}}\cdot v_{u}+\frac{e^{\alpha_{f}}}{e^{\alpha_{u}}+e^{\alpha_{f}}}\cdot v_{f} \end{array}$$

where $\alpha_{u}$ and $\alpha_{f}$ are the learned weights for the users and forums, and $W_{t}$ and $b_{t}\left(t\in \{u,u^{\prime },u^{\prime \prime },f,f^{\prime },f^{\prime \prime }\}\right)$ are the learned weight matrices and biases.

Thus, context representation learning employs user information and forum information to capture the comprehensive context representation, where the context representation not only guides the comment representation learning but also provides background knowledge about the corresponding comments for sarcasm detection.

### 3.5. Decision Learning

Decision learning first combines comment representation and context representation into a unified representation and then employs simple neural networks to predict whether a comment is sarcastic or not. In detail, we first concatenate a refined comment representation $v_{c}$, the last hidden representation $h_{k}$ produced by the Bi-LSTM, and the context representation $v_{con}$. Then, we employ two linear layers with tanh and sigmod activation to compute the probability of the comment belonging to the sarcastic class, which is formulated as follows:

$$\begin{array}{cc} h_{temp} & =concat\left(v_{c},h_{k},v_{con}\right)\\ v_{hidden} & =\tanh\left(W_{hidden}\cdot h_{temp}+b_{hidden}\right)\\ \hat{y} & =\sigma \left(W_{o}\cdot v_{hidden}+b_{o}\right) \end{array}$$

where concat() is the concatenation function, $W_{hidden}$, $W_{o}$, $b_{hidden}$, and $b_{o}$ are learned parameters, and σ is the sigmoid function. Finally, the cross-entropy loss function is employed for training as follows:

$$\mathrm{Loss}=\frac{-1}{t}\sum_{i=1}^{t}\sum_{j=1}^{2}y_{i}^{j}log_{2}\left({\hat{y}}_{i}^{j}\right)$$

where *t* is the number of comments in the training set, $y_{i}$ is the one-hot vector ground truth of the *i*th comment, and ${\hat{y}}_{i}^{j}$ is the predicted probability of belonging to class *j*.

## 4. Experiment

### 4.1. Experimental Data

To evaluate the proposed model, we performed our experiments on the **SARC** (https://nlp.cs.princeton.edu/old/SARC/ (accessed on 1 April 2023)) corpus [30], which was obtained from the social media site Reddit. Reddit contains topic-specific discussion forums, comments, and author details. In the experiment, we employed three variants of the **SARC** dataset, as follows: (1) **Main balanced,** where the dataset is a balanced distribution of both sarcastic and nonsarcastic comments; (2) **Pol balanced,** where the dataset is a subset of the Main dataset, comprising forums associated with the topic of politics; and (3) **Pol imbalanced,** where the dataset is an imbalanced version of the dataset with the topic of politics. We sampled sarcastic comments and nonsarcastic ones in both training and testing sets in a ratio of approximately 20:80. The details of the datasets are shown in Table 1.

### 4.2. Comparison Models

Here, we describe the state-of-the-art methods and baselines that we compared the CSDM with.

- **CBOW** The model employs the continuous bag of words to represent comments and applies a fully connected layer to recognize sarcasm.
- **CNN** The model applies a single CNN on the comment to capture location-invariant local patterns and combines the CNN with a fully connected layer to detect sarcasm.
- **LSTM** The model uses the Bi-LSTM on the comment to capture the long-range dependency and combines the Bi-LSTM with a fully connected layer to detect sarcasm.
- **BERT-FCL** The model [23] uses BERT to represent comments and combines BERT with a fully connected layer to detect sarcasm.
- **SAWS** The model [19] adopts a self-attention mechanism of weighted snippets with a context vector to capture the incongruity of sentence snippets.
- **ADGCN** The model [12] employs a graph convolutional network based on an affective graph and a dependency graph in order to capture the long-range literal sentiment inconsistencies.
- **CUE-CNN** The model [9] learns user embeddings by projecting similar users into nearby regions of the embedding space and combines user embeddings with a CNN to detect sarcasm.
- **CASCADE** The model [14] adopts a hybrid approach with both content and context-driven modeling for sarcasm detection, where user embeddings are used to encode the stylometric and personality features of users.

### 4.3. Training Details

In our experiments, we randomly sampled 20% of the training data as the validation set. For comment representation, we employed fastText to obtain a 300-dimensional embedding for each comment, which was either restricted or padded to 100 words. The dimensionality of the hidden representations in the Bi-LSTM was set to 256. For context representation, ParagraphVector was used to embed each forum as a 100-dimensional embedding and each user as a 100-dimensional embedding. The dimensions of the last two fully connected layers were 256 and 1, respectively. In the training process, the learning rate was 0.001, and the batch size was 32. We used the Adam optimizer to optimize the loss function. The hyperparameters are shown in Table 2. For the other baseline methods, we adopted the parameters used in the original papers. Meanwhile, for the CUE-CNN and CASCADE, we adopted the user embeddings trained in our work for consistency.

### 4.4. Experimental Results

In this section, we compare the proposed model, CSDM, with state-of-the-art networks and baselines in terms of the accuracy and F1 score. The results are shown in Table 3. The experimental results illustrate that the CSDM achieved a better performance than the state-of-the-art networks and the baseline models.

Specifically, our proposed model CSDM obtained improvements of 11.2%, 11.1%, and 5% in terms of the accuracy compared with CASCADE on the Main balanced, Pol balanced, and Pol imbalanced datasets, respectively. We also observed that the CSDM obtained improvements of 11.2% and 9.3% in terms of F1 compared with CASCADE on Main balanced and Pol balanced datasets, respectively. This shows that the sophisticated fusion strategy in the CSDM is useful for capturing latent relationships among comments, users, and forums and balancing the semantic representations among them. However, the CSDM had a worse performance on the Pol imbalanced dataset. This may be because the ratio of nonsarcastic comments to sarcastic comments in the Pol imbalanced dataset affects the recall of the corresponding model.

Compared with CBOW, CNN, LSTM, and SAWS, we observed that our proposed model, CSDM, achieved improvements of around 7.8%, 7.6%, and 2.4% in terms of the accuracy and 7.8%, 7.6% and 26% in terms of the F1 on the Main balanced, Pol balanced, and Pol imbalanced datasets, respectively. This demonstrates that contextual information, including user and forum information, is useful for improving the sarcasm detection performance. In addition, we observed that the LSTM obtained better results than CBOW, CNN, and SAWS, which shows that sequential order information is also helpful for sarcasm detection.

Compared with BERT-FCL, ADGCN, and CUE-CNN, we observed that our proposed model, CSDM, achieved improvements of around 6.1%, 4.4%, and 3.7% in terms of the accuracy and 6.1%, 4.4%, and 1.5% in terms of the F1 on the Main balanced, Pol balanced, and Pol imbalanced datasets, respectively. The above results demonstrate that combing contents with user and forum information is important for the improvement of the sarcasm detection performance. Meanwhile, BERT-FCL, ADGCN, and CUE-CNN use different types of additional information, i.e., common background context, affective commonsense knowledge, and user information, respectively, as contextual information, but they only employ one type of contextual information. Our proposed model employs user information and forum information as contextual information and employs sophisticated context-aware attention to capture the comprehensive comment representation.

Compared with BERT-${\mathrm{FCL}}^{★}$, our proposed model, CSDM, achieved improvements of around 1.5%, −6.7%, and 0.0% in terms of the accuracy and 15%, 112%, and −5.6% in terms of the F1 on the Main balanced, Pol balanced, and Pol imbalanced datasets, respectively. We also observed that the CSDM performed better on the Main balanced dataset, and BERT-${\mathrm{FCL}}^{★}$ performed better on the Pol balanced and imbalanced datasets. This may be because BERT can learn more explicit semantic information from the word representations of a single topic than from the word representations of diverse topics.

Meanwhile, we observed that all methods performed better on the Pol imbalanced dataset than on the other two datasets. This may be because the ratio of sarcastic comments to nonsarcastic comments was higher on Pol imbalanced dataset than on the other two datasets. Furthermore, except for the impact of the Pol imbalanced dataset, BERT-FCL, BERT-${\mathrm{FCL}}^{★}$, and CASCADE performed well on the Pol imbalanced dataset, which may have also been caused by the feature learning strategies of the methods.

### 4.5. Ablation Study

We conducted an ablation study of the proposed model, CSDM, in order to analyze the impacts of various feature components in its architecture on the Main balanced, Pol balanced, and Pol imbalanced datasets. The experiment results are reported in Table 4. CSDM-c, CSDM-u, and CSDM-f are models without context learning, user information, and forum information, respectively.

In Table 2 and Table 3, we can observe that the CSDM-c performed better than the LSTM and SAWS. The CSDM-c employs Bi-LSTM and attention mechanisms to learn comment representations. This shows that the combination of Bi-LSTM and attention mechanisms is important for sarcasm detection, since the combination can obtain the local and global contextual information for comment representations.

In Table 3, we can observe that the variants of the CSDM do not perform as well as the CSDM. First, the performance of the CSDM-u is relatively poor compared with the other models. This may be because forum information is very general and abstract, so it provides little useful information. Then, compared with the CSDM-u and CSDM-c, the CSDM-f performs relatively well. This shows that user information can indicate the sarcastic tendencies of users, which is important for sarcasm detection. Meanwhile, compared with the CSDM, the performance of the CSDM-c is poor, which demonstrates that contextual information is helpful for capturing additional information for sarcasm detection. Lastly, the CSDM performs the best among these models. This may be for the following reasons: (1) both comment and contextual information are adopted in order to comprehensively understand the sarcastic tendencies of users; and (2) a context fusion mechanism is used to capture the latent complex relationship between users and forums.

### 4.6. Case Study

In this section, we sample some sarcastic comments to showcase the mechanism of the comment representations presented in Table 5 with the corresponding posts as the context. The comment representation indicates the content features of comments by integrating contextual information with the Bi-LSTM and attention layers in order to capture the long-dependency relationships between words and key parts of sentences in the comments. Examples of sarcasm expression demonstrate that the comment representation determined by capturing relationships between words is highly effective for identifying the incongruity information in sentences. For example, in the comment *“well yeah, cuz they ‘re bortin’ all tha babees”*, we notice that the words *“cuz”* and *“all”* have the highest attention weights. Meanwhile, in the sentences *“yeah, who would have figured that a rich billionaire wasn’t going to be an advocate for the working class”*, *“don’t you know, this is totally normal for a president to have”*, and *“but both sides are exactly the same”*, the words *“would”*, *“figured”*, *“totally”*, *“but”*, and *“sides”* obtain relatively high attention weights. The most attended words provide the opposite sentiments in sarcastic expressions.

Therefore, the comment representations identified by integrating contextual information could capture the incongruity information of sentences, which could provide a strong indicator of the appearance of sarcasm.

### 4.7. Error Analysis

Below, we report a couple of sarcasm samples from the Pol balanced dataset presented in Table 6 which obtained incorrect prediction results compared with the ground truth of the samples. The above bad cases were used for an error analysis of our proposed model in order to understand the circumstances under which our proposed model will fail.

First, our model neglected the incongruity expressions between *“$3.76B”* and *“small price”* for the first example, which may be because the model lacks common background knowledge of *“small price”*. Meanwhile, the model did not understand the word *“waterboarded”* for the second example, which meant that the model captured the incongruity expressions between *“waterboarded”* and *“raised”*. For the third example, the model ignored the background situation for the phrase *“against freedums”*. For the fourth example, the meaning of the post and the comment is consistent, but the phrase *“would be”* may disturb the judgment of the model. For the fifth example, the phrase *“only if”* may also disturb the judgment of the model. For the last example, the phrase *“probably not the best time”* may affect the results. In all, the error analysis demonstrates that the implicit semantic information and uncertainty information of words and some question formats can cause poor performance of the proposed model.

## 5. Conclusions

In this work, we proposed a Contextual Sarcasm Detection Model based on diverse contextual cues retrieved from external discourses used in online social media. The proposed model employs context-aware attention and a user-forum fusion network to generate a comprehensive fused representation. First, we employed a Bi-LSTM encoder to capture the sentence composition information and we adopted context-aware attention to obtain a refined comment representation with contextual information. Then, we employed a user-forum fusion network to capture the sarcastic tendencies of users and the background knowledge about comments by integrating user information and forum information. Experimental results on the large Reddit corpus demonstrate that the proposed method achieves a significant performance improvement over state-of-art textual sarcasm detection methods in terms of standard evaluation metrics including the F1 score and accuracy. Furthermore, the methodology could be applied to languages other than English in future work.

## Figures and Tables

**Table 1:** Statistics of the experimental data.

Dataset	Training		Testing	
	Nonsarcastic	Sarcastic	Nonsarcastic	Sarcastic
Main balanced	77,351	77,351	32,333	32,333
Pol balanced	6834	6834	1703	1703
Pol imbalanced	37,941	9485	21,070	2341

**Table 2:** Training details of the experiments.

Parameter	Value
Maximum sequence Length	100
Batch Size	32
Learning rate	0.001
Dimensions of comment embedding	300
Dimensions of user embedding	100
Dimensions of forum embedding	100

**Table 3:** Comparison of the CSDM with state-of-the-art networks and baselines on multiple versions of the SARC dataset. The BERT-FCL employs the pretrained language model BERT released by Google. The BERT-${\mathrm{FCL}}^{★}$ was used to fine-tune BERT on our training dataset.

Models	Main Balanced		Pol Balanced		Pol Imbalanced	
	Accuracy	F1	Accuracy	F1	Accuracy	F1
CBOW	0.60	0.60	0.60	0.60	0.80	0.44
CNN	0.63	0.63	0.65	0.65	0.81	0.53
LSTM	0.50	0.45	0.50	0.43	0.80	0.44
BERT-FCL	0.65	0.65	0.57	0.56	0.73	0.71
BERT-${\mathrm{FCL}}^{★}$	0.68	0.60	**0.75**	0.33	**0.83**	0.71
SAWS	0.64	0.64	0.59	0.59	0.80	0.54
ADGCN	0.62	0.62	0.65	0.65	0.79	0.66
CUE-CNN	0.59	0.57	0.67	0.67	0.80	0.44
CASCADE	0.62	0.62	0.63	0.64	0.79	**0.88**
CSDM	**0.69**	**0.69**	0.70	**0.70**	**0.83**	0.67

**Table 4:** Comparison with variants of the proposed CSDM network. CSDM-c, CSDM-u, and CSDM-f are models without context learning, user information, and forum information, respectively.

Models	Main Balanced		Pol Balanced		Pol Imbalanced	
	Accuracy	F1	Accuracy	F1	Accuracy	F1
CSDM-c	0.67	0.67	0.66	0.66	0.80	0.66
CSDM-u	0.66	0.66	0.68	0.68	0.80	0.65
CSDM-f	0.68	0.68	0.69	0.69	0.80	0.68
CSDM	0.69	0.69	0.70	0.70	0.83	0.67

**Table 5:** Attention visualization. For each sample, the sentence demonstrates the original comment and the corresponding attention weights generated from the CSDM; the darkness of the background varies according to the value of the corresponding attention weight.

Post	Comment
Analysis | States with more Planned Parenthood clinics have fewer teen births and sexually transmitted diseases	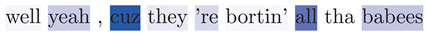
Alec Baldwin: Trump “An Enemy of the Working Class”	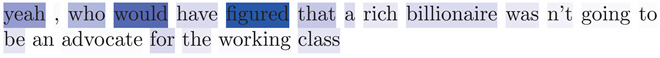
No wonder he won’t release his tax returns; Trump has business ties to at least 10 alleged former Soviet criminals, report claims	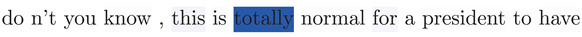
Net Neutrality Is Trump’s Next Target, Administration Says	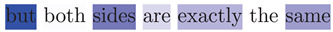

**Table 6:** Comment samples of error analysis.

Post	Comment	Ground Truth	Prediction
Bathroom bill’ to cost North Carolina $3.76B	small price to pay for keeping their women and female children safe	Sarcasm	Nonsarcasm
Ted Koppel tells Sean Hannity he is bad for America	come on remember all the money he raised for the charity being waterboarded?	Sarcasm	Nonsarcasm
Republican lawmakers introduce bills to curb protesting in at least 18 states	but the democrats are against freedums	Sarcasm	Nonsarcasm
Border wall ask: $1 billion for 62 miles	who knew tall concrete walls would be expensive?	Nonsarcasm	Sarcasm
Star Wars: US Must Prep for Space Battles, Commander Says	only if the space ships run on coal	Nonsarcasm	Sarcasm
Scarlett Johansson May Run for Office, Isn’t Concerned About Boycotts	probably not the best time for her to be speaking on politics when her newest movie features her as a whitewashed version of an Asian heroine	Nonsarcasm	Sarcasm

## Data Availability

Publicly available datasets were analyzed in this study. These data can be found here: (https://nlp.cs.princeton.edu/old/SARC/ (accessed on 1 April 2023)).
